# Editorial

**Published:** 1861-06

**Authors:** 


### Editorial.

### POSTPONEMENT.

We see from various sources, that postponement is the order of the day. We learn by the medical journals that the meeting of the American Medical Association is postponed for one year ; the same is true of many other meetings and associations. This has been deemed advisable in consequence of the very peculiar circumstances in which our country is placed at present. Men who have heretofore coöperated in these various enterprises, are now thrown into such positions as would render their meeting and harmonizing even in scientific pursuits impracticable. There are other reasons, however, of more force than this. The minds of scientific and professional men have been so much diverted from their specialties, and in common with others, so entirely carried away by the current events of the day, as to be altogether unfitted for scientific research and investigation, so that evidently the preparation that they would have made in view of these meetings, will not be accomplished, and hence little else would be done than mere routine, business transactions. This would be simply running the machinery without accomplishing any work; and again, financial matters are in such a disturbed condition, that most persons prefer to *spend* no more money than is necessary for actual current living expenses.

These objections stand as fully in the way of the meetings of the National Dental Association and Dental Convention, as of any other bodies. In regard to the National Association, there was a large amount of work designated to be accomplished at the next meeting ; very much of this, we fear, will not be accomplished. Several have written to us, saying they could not perform the work assigned them, and we have seen or heard of no one who says he is able to perform his work.

We say this with all due respect for their ability to do what they intended, were the circumstances not so forbidding. In this matter, we desire that the best should be done. If the committee of arrangements and the officers of the American Dental Association think it best that the meeting should be held at the appointed time, let us go vigorously to work, and prepare for it. But it is our firm conviction, that were this resolution formally made, entered into, and agreed upon, that the meeting would be a failure, in two respects, *but few would go, and those who did would not be prepared to do anything.* T.

---

### INTERNAL DECAY.

We see, in the discussions of the Kentucky State Dental Association, that in considering the subject of dental caries, the "internal decay" question came up. If any one will consider the manner in which decomposition takes place, and then give a rational theory for internal decay of the teeth, he will place the dental profession under great obligation, and under still greater obligation if he will prove his theory by facts.

The decomposition of dentine is effected by the operation of chemical agents, having sufficient force to destroy the integrity of either the calcarious or animal constituents of the dentine. The vitality and circulation of dentine is of so low a grade, that it is not susceptible of ulceration or spontaneous decomposition, even when the vitality is destroyed ; much less while vitality exists, for it always resists decomposition.

There is no element in the dentine that can break down the structure, nor is the absorption and circulation sufficient to carry away the fragments, even if it was broken up. Dr. Rogers remarks,

"That two causes produce decay, chemical and inflammatory;" he further remarks, "Inflammatory causes must for the most part be presumed." If it is merely a presumption, why speak of it afterward as a truth that has been demonstrated? He refers to a case, in which there was sensitiveness and pain of the dentine, "without apparent decay;" "after three years, decay appeared, thus showing that decay had commenced inside." We can not conceive how this proves the existence of internal decay. The sensitiveness and pain was from an inflamed condition of the dentine, beginning upon its surface, by the influence of irritating agents, that were not as yet able to produce decomposition or decay, until they had attained increased energy and reduced the resisting power to that point at which an attack was possible. Sensitiveness of intact dentine many a time exists, but we have not before heard it suggested as an evidence of internal decay.

The fact that teeth sometimes decay under fillings, is perhaps better explained by the imperfection and inefficiency of the fillings, than upon the internal decay principle. Dr. Nourse "has seen several cases, showing clearly to his mind that decay is frequently produced by inflammation." What kind of cases it is, that will show clearly to a man's mind that which does not exist, we are rather at a loss to know. Dark spots under the enamel does not prove internal caries very clearly to our obtuse mind. We wait with patience, but considerable interest, for the rationale of internal caries produced solely by inflammation. T.

### NEW CAUSTIC HOLDER.

We have received a caustic holder from Geo. Tiemann & Co., of New York, which is a superior thing in its way. Dr. Edwards, in speaking of it, says: "The three requisites for a good caustic holder are—First, indestructibility; second, facility of charging; and third, protection of the caustic when not in use or being carried. These points are all obtained in this holder. It consists of a tube, divided longitudinally, with a screw thread cut in the bore, to give a rough hold on the caustic. The halves of the tube are attached to the blades of a light spring forceps in such a way as to be brought accurately together; by pressure upon the blades, the tube is opened that the caustic may be introduced ; it is then hel firmly in the tube while being used, after which it may be dropped entirely into the tube for safe keeping while not being used or being carried. The one we have is some ten inches long, much longer than is necessary for the dentist's use. We would suggest that the manufacturer make them about four inches long for the dentist's use.

The instrument is so arranged that the caustic can not touch the steel or anything that it can corrode.

Every one who uses nitrate of silver should have one of these instruments. T.

---

### A DRILL SHIELD.

This is a neat little instrument, made of silver plate, in the form of a tube about two inches long, and two lines in diameter, or large enough to receive freely the ordinary bur drill shaft. There is upon it a sliding collar, to which is attached a flat arm, about half an inch long, at right angles with the tube ; this arm is designed to rest against the finger of the left hand of the operator, or upon the teeth of the patient, to prevent the tube rotating with the drill.

The object of the instrument is to prevent the shaft of the drill from abrading the lips or cheeks of the patient, while being used ; it accomplishes that object well. It was invented by D. G. Hussey, and is for sale by J. T. Toland. T.

---

### "HUMAN KNOWLEDGE" AND "IMPARTATION."

Two articles, with the above titles respectively, have lately appeared in the Register. They are both worthy of the reader's careful attention. They have nothing in common—are not a bit alike—unless it be that both are long articles. One is clear, demonstrntive, *instructive*, and convincing It appears to come from a mind that has almost mastered its subject. The other is like a landscape view that ends away off in the blue distance, and leaves the soul unsatisfied, because it knows there is *land beyond* that it would like to see. But the soul, unsatisfied as it is, looks and longs, and longs and looks again, thrilled with the delight of its own unsatisfiedness. This article is entitled "IMPARTATION." Don't ask us what it is about. We don't know—don't want to know. All we read and re-read it for, is for the luxury of seeing a great big, impatient, overflowing soul, leaping, climbing, and straining itself to get beyond its present confined sphere of existence. No better evidence of the immortality of the soul is needed. It almost says, "I pray thee, let me go over and see that good land." Here we know only in part, but there— W.

### PERSONAL.

THE readers of the *Register* may have noticed that we bestowed no labor on the last two numbers. They have not suffered, unless from curiosity to know what has become of us. Well, we are alive, "and alive like to be," if cheerfulness and good appetite signify anything. But you all know that bad colds, fevers, and most other diseases, fasten on the weakest organs of the system. An attack of fever made a survey of our system, and where should it conclude to settle but on the brain? Finding its domicil "empty, swept and garnished," it claimed "squatters' rights," and held on to its possession, till it was beleaguered and bombarded by a detachment of Esculapian artillery. After the surrender, the engineers of the said artillery regarded the fort as unfit for use, till repaired and strengthened. And now, reader, you know it all. We couldn't write without thinking, couldn't think without a brain, and the "doctors" wouldn't let us use our brain, and hence our long silence. W.

### "WHOLLY GIVEN TO IDOLATRY."

EPHRAIM was joined to his idols; and he was *let alone.* In like manner, the Pennsylvania Association of Dental Surgeons seems to be joined to "amalgam;" and, as far as the Society and its members are concerned, we are disposed to let them alone. We would let them *alone,* if we could; but we can't. They are not *alone,* for all the drones in the profession, all the ignorant and unskilled, all the unprincipled, all the lazy, and all the stupid members and appendixes of our profession are with them. The members of the Pennsylvania Association are clever fellows—all; but, shade of Crawcour, brethren, what company you keep!

While leaving these brethren to enjoy the society of these comrades of their own choosing, we may, some time when we feel like it, say a little about some of the positions advanced at the April 9th meeting of the association. If we say anything, it will be from a sense of duty to our younger professional brethren, and not, certainly, from a desire for controversy on this hackneyed subject.

W.

### REPORT OF DISCUSSIONS.

Our report of the discussions of the late meeting of the Mississippi Valley Association falls far short of justice to the society, or to its members as individuals. The report was written out during the premonitory stages of an attack of fever. It had to be written under such circumstances, or not at all. W.

### A NEW DENTAL COLLEGE

Is organized at New Orleans. We have a personal acquaintance with two members of the Faculty; and, unless we are deceived in our reckoning, they will not be found wanting. The Faculty is, to a good degree, composed of alumni of the Ohio Dental College, and, therefore, should be competent, and ought to succeed. There is room for a dental college in New Orleans. W.

### "IN ALL THY WAYS ACKNOWLEDGE GOD."

The meetings of the Kentucky State Dental Association were opened with prayer, in accordance with the worthy example set by the late President of the Mississippi Valley Association, Dr. Atkinson. It is certainly becoming for those in search of wisdom to look to the Source of all wisdom—for those in pursuit of knowledge to recognize their dependence on the Author of all knowledge. "Ask and ye shall receive." W.

